# Antimicrobial Activity of *Aspergillus* sp. from the Amazon Biome: Isolation of Kojic Acid

**DOI:** 10.1155/2022/4010018

**Published:** 2022-05-17

**Authors:** Josy Caldas Rodrigues, Weison Lima da Silva, David Ribeiro da Silva, Carolina Rabelo Maia, Clarice Virginia Santos Goiabeira, Haile Dean Figueiredo Chagas, Gigliola Mayara Ayres D'Elia, Gleica Soyan Barbosa Alves, Viviane Zahner, Cecilia Veronica Nunez, Ormezinda Celeste Cristo Fernandes

**Affiliations:** ^1^Leônidas and Maria Deane Institute-ILMD/Fiocruz Amazônia, Terezina street, 476, Adrianópolis, Manaus, Brazil; ^2^National Institute of Amazonian Research, André Araujo Avenue, 2936, Petrópolis, Manaus, Brazil; ^3^Exact Sciences and Technology Institute-ICET/UFAM, Nossa Senhora Do Rosário Street, Tiradentes, Itacoatiara, Brazil; ^4^Oswaldo Cruz Institute/IOC/Fiocruz, Brasil Avenue, 4365, Manguinhos, Rio de Janeiro, Brazil

## Abstract

The antimicrobial potential of *Aspergillus* sp., isolated from the Amazon biome, which is stored at the Amazon Fungi Collection-CFAM at ILMD/FIOCRUZ, was evaluated. The fungal culture was cultivated in yeast extract agar and sucrose (YES) for cold extraction of the biocompounds in ethyl acetate at 28 °C for 7 days in a BOD type incubator. The obtained extract was evaluated for its antimicrobial activity against *Candida albicans* and Gram-positive and negative bacteria by the “cup plate” method and the determination of the minimum inhibitory concentration (MIC) by the broth microdilution method. The extract was subjected to thin layer chromatography (TLC) and fractionated by open and semipreparative column chromatography. The fractions of interest had their chemical constituents elucidated by nuclear magnetic resonance and mass spectrometry. The elucidated molecule was evaluated for cytotoxicity against the human fibroblast strain (MRC5). The extract presented inhibitory activity against both Gram-positive and negative bacteria, with the range of inhibition halos from 5.3 to 14 mm in diameter and an MIC ranging from 500 to 15.6 *μ*g/mL. Seventy-one fractions were collected and TLC analysis suggested the presence of substances with double bond groups: coumarins, flavonoids, phenolic, alkaloids, and terpenes. NMR and MS analyses demonstrated that the isolated molecule was kojic acid. The results of the cytotoxicity test showed that MRC5 cells presented viability at concentrations from 500 to 7.81 *μ*g/mL. The kojic acid molecule of *Aspergillus* sp., with antibacterial activity and moderate toxicity at the concentrations tested, is a promising prototype of an alternative active principle of an antimicrobial drug.

## 1. Introduction

The search for biomolecules of fungal origin with antimicrobial activity has intensified in recent years due to the increase in resistant microorganisms and the absence of drugs to fight them [1–3]. Among the microorganisms resistant to some antimicrobial agents, we can highlight methicillin-resistant *Staphylococcus aureus* (MRSA) and *β*-lactamase-producing *Escherichia coli*, which are no longer associated exclusively with hospitals, but are emerging in the community [4, 5]. According to data from the World Health Organization (WHO), if nothing is done to prevent the spread of antimicrobial resistance on the planet, the deaths of people caused by resistant microorganisms could exceed 10 million per year by 2050 [6, 7]. However, researchers and physicians warn that, due to the COVID-19 pandemic and the rampant use of antibiotics to prevent coinfections, this scenario of deaths by super-resistant microorganisms can become even worse [8, 9].

In this context, species of the genus *Aspergillus* stand out for being known as producers of several natural substances with varied structures and with considerable biological actions of great biotechnological importance [10, 11]. Studies on the antimicrobial potential of *Aspergillus* species found in the soil of the Amazon region have been growing in recent years, providing a potential source of new chemical substances with different biological actions that can become promising bioactive prototypes [12].

Considering the need for further research on the search for active molecules and the possibility of obtaining new products from species of the genus *Aspergillus*, very common in terrestrial ecosystems in the Amazon, this research aimed to verify, *in vitro*, the antimicrobial activity of *Aspergillus* sp., isolated from the Amazon biome and stored in the Amazon Fungi Collection-CFAM and chemically identify the potential molecules for the tested biological activity.

In order to develop the Amazon region, biotechnology is one of the main areas that can provide growth in a sustainable way. Our research group focuses on the bioprospection of soil fungus. Thus, this work focused on performing the bioprospecting of *Aspergillus* sp. isolated from the Amazon soil biome and stored in the Amazon Fungi Collection-CFAM in order to perform bioassays for the antimicrobial activity of the extract and cytotoxicity of the isolated molecule(s).

## 2. Materials and Methods

### 2.1. Study Location

The selection of strains, crude extract obtainment, and the biological activity of this work were carried out at the Multiuser Laboratory in Health/Mycology at the Leônidas and Maria Deane Institute (Instituto Leônidas *e* Maria Deane) – ILMD/FIOCRUZ Amazônia. The chemical elucidation of molecules with biological activity was carried out at the Laboratory of Bioprospecting and Biotechnology of the National Institute for Research in the Amazon (Instituto Nacional de Pesquisas da Amazônia, INPA).

### 2.2. Microorganisms

For this study, the fungus *Aspergillus* sp. stored in the Amazon Fungi Collection (CFAM), from the Leônidas and Maria Deane Institute - ILMD/FIOCRUZ Amazônia was selected. The test microorganisms were provided by the Amazon Bacteria Collection (CBAM), Amazon Fungi Collection (CFAM), and Collection of Microorganisms of Medical Interest at INPA.

### 2.3. Reactivation of Selected Filamentous Fungi

The selected fungi were reactivated in a Petri dish containing malt extract agar (MEA) and incubated at 28 °C for 7 days in a BOD type incubator [13].

### 2.4. Extraction of Secondary Metabolites

The fungal culture was grown in an Erlenmeyer flask (500 mL) containing 200 mL of sucrose and Czapek yeast extract agar (YES) at 28 °C. After seven days of growth, the metabolites were cold extracted in 200 mL of ethyl acetate (EtOAc) P.A (SYNTH). The EtOAc extract obtained after 48 hours of extraction was filtered and subjected to concentration in a rotary evaporator (SCILOGEX RE 100-Pro) and further used to determine the antimicrobial activity [12].

### 2.5. Biological Assays

#### 2.5.1. Evaluation of the Fungi for Their Antimicrobial Potential

#### 2.5.2. Determination of Antimicrobial Activity

The EtOAc extract was evaluated in terms of antimicrobial activity against *Candida albicans* CFAM 1186, *Staphylococcus aureus* CBAM 324, *Staphylococcus aureus* ATCC 27853, *Staphylococcus aureus* ATCC 43300 (MRSA), and *Escherichia coli* CBAM 0002 by the cup plate method by agar diffusion.

Bacteria and yeast were reactivated in Petri dishes (10 mm × 90 mm) containing Mueller–Hinton agar and Sabouraud agar, respectively. The cultures were kept at 37 °C for 24 hours (bacteria) and 48 hours (yeast). From these cultures, a cell suspension of similar concentration to column n°1 of the McFarland scale was prepared, and from these, 100 µL were removed to be seeded on the surface of the Petri dish containing Müeller–Hinton agar or Sabouraud agar. In these cultures, three wells of 5 mm in diameter were made and 150 µL of fungal extract at a concentration of 2 mg/mL was added to each well. The extracts were solubilized in sterile distilled water and 4% dimethylsulfoxide (DMSO). The plates were placed in an incubator at 37 °C for 24 to 48 hours. As a standard, vancomycin and itraconazole were used at a concentration of 2 mg/mL (MERCK, Germany). The antimicrobial activity was evaluated by measuring the inhibition halo in mm against the test microorganism. The entire test was performed in triplicate [12].

### 2.6. Determination of the Minimum Inhibitory Concentration (MIC) by the Broth Microdilution Method

The determination of the minimum inhibitory concentration of the obtained EtOAc extract was performed according to the broth microdilution technique using 96-well sterile polystyrene plates [14]. The extract, with an initial concentration of 2 mg/mL, was solubilized in sterile distilled water and 4% dimethylsulfoxide (DMSO), and the inoculum of test microorganisms was prepared in medium by suspension of the cultures and adjusted to column° 1 of the McFarland scale. To reveal the results, a 1% solution of triphenyltetrazolium chloride (TTC) in sterile distilled water was prepared. The microplates were incubated in a BOD type incubator at 37 °C for 24 to 48 hours. After the incubation period, the reading was performed, checking for the growth of the microorganism, indicated by the purple color, and inhibition, indicated by the absence of the purple color. MIC was determined as the lowest concentration of fungal extract capable of preventing microbial growth. The extract concentrations used in this study ranged from 1000 to 15.6 *μ*g/mL. All tests were performed in triplicate.

### 2.7. Chemical Prospection

#### 2.7.1. Chromatographic Fractionation of the Extract and Isolation of the Substance

The fractionation of the EtOAc extract of *Aspergillus* sp. (298 mg) was carried out using the open column chromatography (CC) technique and as stationary phase, silica gel 60 (230–400 mesh-Merck) was used in a 1 : 80 proportion (hx Ø = 54.5 × 1 cm). Using gradient mode elution systems, CHCl_3_/MeOH (95 : 5, 9 : 1, 85 : 15, 8 : 2, 5 : 5, and 100% MeOH).

The fractionation of the EtOAc extract resulted in 71 fractions that were further analyzed by thin layer chromatography (TLC). After the analysis, the fractions that showed similarity were pooled, resulting in 4 fractions (3, 4–8, 9–11, and 16–18), and from these, the 16–18 fractions were selected to proceed with the fractionation.

Fractions 16–18 (109 mg) were fractionated once more by open column chromatography (CC), using as stationary phase silica gel 60 (230–400 mesh-Merck) in the proportion of 1 : 80 (hx Ø = 17.5 × 1 cm). Elution systems in gradient mode, CHCl_3_/acetone (9 : 1, 8 : 2, 7 : 3, 1 : 1, acetone and 100% MeOH) were used, resulting in 67 fractions which were analyzed by TLC. After the analysis, fractions 31–38 (69 mg) were subjected to purification in high-performance liquid chromatography (HPLC) in a semipreparative column (5 µm, C18 column, 100 A, 250 mm × 10 mm, injection volume 50 µL, flow rate 4.7 mL/minute) using methanol 25% pH 5 isocratic mode.

### 2.8. Chromatographic Analysis of Fractions by TLC

The collected fractions were analyzed by thin layer chromatography (TLC), using aluminum-supported silica sheets impregnated with a 254 nm fluorescein indicator (MERCK). Chloroform (CHCl_3_)/methanol (MeOH) 9 : 1 was used as the mobile phase in order to separate the chemical constituents from the fractions. Physical developers were used: ultraviolet light 365 and 254 nm, and chemical developers included Ce (SO_4_)_2_, anisaldehyde, FeCl_3_, NP-PEG, and Dragendorff reagent [15, 16].

### 2.9. Spectroscopic and Spectrometric Analysis

The analyses were carried out at the Analytical Center of the Thematic Laboratory of Chemistry of Natural Products–LTQPN of INPA.

The structural identification of the isolated substance was made through the analysis of the nuclear magnetic resonance (NMR) of hydrogen and carbon thirteen spectra (^1^H and ^13^C, one and two-dimensional). And by mass spectrometry (Model MicroTOF-Q Spectrum, Bruker Daltonics), in LC/MS mode, electrospray, operating in positive and negative mode.

### 2.10. *In Vitro* Cytotoxicity Assay

The cytotoxicity test was performed with the substance isolated in the laboratory of the RPT11H-Bioassays of Biotechnological Compounds platform at the Leônidas and Maria Deane Institute (ILMD). The substance was solubilized in 100% DMSO, with a final concentration of 0.01% in the well. And it was tested at eight concentrations: 1.000, 500, 250, 125, 62.5, 31.25, 15.62, and 7.81.

For this assay, the MRC5 human fibroblast strain was used, with culture conditions adapted for the laboratory of the RPT11H-Bioassays of biotechnological compounds platform at the Leônidas and Maria Deane (ILMD) Institute. The MRC5 strain was grown in Dulbecco's modified eagle medium (DMEM) (Gibco), supplemented with 10% inactivated fetal bovine serum (Gibco), and penicillin antibiotic (50 *μ*g/mL) and maintained in a CO_2_ incubator at 37 °C with an atmosphere containing 5% CO_2_. All tests were performed in triplicate.

The assays were determined by the Alamar blue method, according to Ahmed et al. [17], in order to analyze the cell viability of the MRC-5 strain cells in the presence of different concentrations of the tested substances. Cells were plated at a concentration of 1.0 x 10 cells/well in a 96-well plate, and kept for 24 hours in a 5% CO_2_ incubator at 37 °C. After this time, the substances at the concentrations mentioned above were added, and the plates were kept in a CO_2_ incubator at 5% CO_2_ at 37 °C for 72 hours. Then, 10 *μ*L of 0.4% resazurin (diluted 1 : 20) was added to each well, and the Alamar blue® (Sigma-Aldrich, Brazil) metabolization time of 2 hours was awaited. Fluorescence was monitored in a microplate reader (GloMax® Explorer) emission wavelength 580–640 nm and excitation 520 nm. Cell growth was used as a positive control; as a negative control, 0.1% DMSO was used. As a drug control, doxorubicin was used and tested at the same concentrations of substances. The percentage of cell viability was calculated according to the formula: %viability = Ft *x* 100/Fb, where Ft= (cell fluorescence + medium + substance + resazurin) and Fb= (cell fluorescence + medium + resazurin).

## 3. Results and Discussion

### 3.1. Biological Assays

#### 3.1.1. Extract Evaluation for Its Antimicrobial Potential by the Cup Plate Method

In the result of antimicrobial activity by diffusion assay, the EtOAc extract of *Aspergillus* sp. showed inhibitory activity against *E. coli* CBAM 0002, *S. aureus* CBAM 0324, *S. aureus* ATCC 27853, and *S. aureus* ATCC 43300 (MRSA) with inhibition halos ranging from 7 to 14 mm in diameter (Table 1).

Based on the data obtained, it can be seen that the EtOAc fungal extract showed promising inhibitory actions against four of the six tested microorganisms, however it was more active against Gram-positive bacteria. Al-Fakih and Almaqtri [18] report that molecules and extracts isolated from *Aspergillus* spp. exhibit greater antibacterial activity against Gram-positive than Gram-negative bacteria.

The results regarding the inhibition of Gram-positive bacteria obtained in this work corroborate with the data obtained by *Lima* [19] when evaluating the bioprospection of antimicrobials produced by fungi from the Amazonian soil, where the *Aspergillus* fungus (H63) filtrate, tested by him, presented an 8 mm inhibition halo for *S. aureus* ATCC 43300 (MRSA) and *S. aureus* ATCC 25923. However, it differs from the result obtained against Gram-negative bacteria since *E. coli* did not show sensitivity to the *Aspergillus* fungus filtrate (H63) tested by *Lima* [19]. It is worth highlighting that the *Aspergillus* sp. extract tested in this work presented higher halos compared to those obtained in the study by *Lima* [19] against *S. aureus* ATCC 43300 (MRSA).

Studies carried out by Souza et al. [20] and Moraes Neto et al. [21] also report the sensitivity of Gram-positive bacteria to extracts of filamentous fungi. According to Moraes Neto et al. [21], this sensitivity may be related to the cell wall of Gram-positive bacteria being more compromising than the cell wall of Gram-negative bacteria, as they have an outer membrane that hinders the action of certain antibiotics. It is known that the Gram-positive bacterial cell wall contains peptidoglycans, teichoic, and teichuronic acid. On the other hand, Gram-negative bacteria contain peptidoglycans, lipopolysaccharides, lipoproteins, phospholipids, and proteins in their cell walls [22].

Prado et al. [23] also showed the selectivity of the antimicrobial activity of *Aspergillus* compounds against test microorganisms and suggest that this selectivity may also be related to the method, the conditions for extracting the compounds, and the form of cultivation of the fungi.

### 3.2. Determination of the Minimum Inhibitory Concentration (MIC) by the Broth Microdilution Method

Using the broth microdilution technique, it was possible to observe that the extract was able to inhibit the growth of test microorganisms at different levels (Table 2). For *E. coli* CBAM 0002 growth was inhibited at a concentration of 15.6 *μ*g/mL and *S. aureus* ATCC at 31.2 *μ*g/mL. *S. aureus* ATCC 43300 (MRSA) growth was inhibited at a concentration of 250 *μ*g/mL and *S. aureus* CBAM 0324 at 500 *μ*g/mL.

There is no consensus in the literature for the MIC of natural products when compared to conventional antimicrobials. However, some authors report that MICs lower than 1 mg/mL can be considered as having antimicrobial potential.

Holetz et al. [24] and Reagasini et al. [25] rank MIC values less than 100 *μ*g/mL as potent activity, 100–500 *μ*g/mL as moderate activity, and 500–1000 *μ*g/mL as weak activity. Therefore, following the standards of these authors, the present work presented values ranging from potent to moderate activity, showing the potential of *Aspergillus* sp. for research and development of new antimicrobial substances.

## 4. Chemical Prospection

### 4.1. Chromatographic Analysis of Fractions by TLC

Fractions 3 to 18 when visualized at wavelengths of 254 nm showed the presence of chromophores, resulting from the absorption of substances with conjugated double bonds. At wavelengths of 365 nm, it was possible to observe blue fluorescence. Based on the fluorescence staining, the presence of classes of coumarins, flavonoids, and alkaloids containing aromatic rings can be suggested [26, 27].

The FeCl_3_ stains showed dark brown spots in the center and on the top of the chromatographic sheets, indicating the presence of phenolic substances. Development with Dragendorff reagent showed the presence of orange stains, indicating the presence of classes of nitrogen molecules (alkaloids). When revealed with anisaldehyde and ceric sulfate, purple stains were observed on the top and middle of the chromatographic sheets, suggesting the presence of steroids or terpenes.

As observed in the TLC analysis, the *Aspergillus* sp. EtOAc extract produced a variety of molecules from different classes, corroborating what is described in the literature for the genus to which it belongs. The genus *Aspergillus* has more than 350 cataloged species, which are known to produce a vast number of bioactive compounds that are beneficial to human health, such as alkaloids, coumarins, terpenes, and lignins [18, 28, 29]. Terpenes, flavonoids, and alkaloids are chemical classes much sought after by researchers because they have several pharmacological activities such as antiviral, antitumor, anti-inflammatory, antimicrobial, antioxidant, and hormonal activities, among others [30–32].

Thus, the *Aspergillus* sp. EtOAc extract showed promise for the development of drugs, presenting molecules with a wide variety of chemical classes already known to be antimicrobial producers.

### 4.2. Purification and Structural Identification of Kojic Acid

From the purification of fractions (31–38), it was possible to obtain a substance whose characteristic was defined as white needle-shaped crystals with a yield of 5 mg. The substance had a retention time of 3.76 minutes, with absorption at wavelengths of 280 nm and 254 nm.

The sample was solubilized in deuterated dimethylsulfoxide (DMSO-*d*_6_) using tetramethylsilane (TMS) as an internal standard.

Analysis of the ^1^H NMR spectrum indicated the presence of five signals, three hydrogens in the aromatic region; *δ* 6.33 and 8.02 both singlets (s) with integral for 1H and at *δ* 9.06 referring to hydroxyl (OH); the other two signals in the aliphatic region; *δ* 4.29 s, integral for 2H and at *δ* 5.67 referring to hydroxyl (OH). The two-dimensional spectra indicated the presence of 6 carbons. Mass spectrum analysis showed a peak with *m/z* 142.11. To corroborate the identification of the molecule, the information about the spectra was compared in the literature, as shown in Table 3.

**Figure 1:**
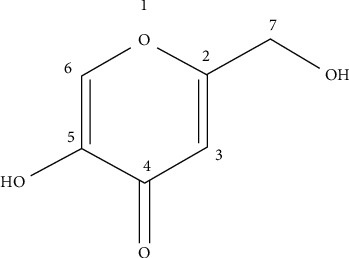
Chemical structure of kojic acid [5-hydroxy-2-(hydroxymethyl)-4-pyrone].

After analyzing the one- and two-dimensional spectra (Table 3) and mass spectrometry, together with the comparison with the literature, the isolated substance was identified as [5-hydroxy-2-(hydroxymethyl)]-4-pyrone, with the name kojic acid.  Figure 1 shows the chemical structure of kojic acid.

Table 3 presents the information obtained through the one- and two-dimensional spectra of ^1^H and ^13^C, comparing it with data available in the literature. In Figure 1, it is shown the kojic acid structure with the chemical shifts of carbons and hydrogens.

### 4.3. *In Vitro* Cytotoxicity Assay

In the cytotoxicity assay, the kojic acid substance isolated from *Aspergillus* sp. had a mean viability of 69.98% at an initial concentration of 1.000 µg/mL. At a concentration of 250 µg/mL, the sample presents a percentage of viability above 70%. According to the results obtained in the cytotoxicity test, the molecule showed satisfactory MRC5 cell viability at concentrations from 500 to 7.81 *μ*g/mL, showing moderate toxicity at the concentrations tested, which makes kojic acid a promising molecule as an antibacterial prototype.

Kojic acid [5-hydroxy-2-(hydroxymethyl)]-4-pyrone is an organic acid that was discovered in Japan by Saito in 1907 from the fungus *Aspergillus oryzae* cultivated on rice [34]. Since the beginning of its discovery, this natural substance has been widely studied due to its various uses in various sectors such as food, cosmetics, medicine, agriculture, and others, resulting in great demand for this metabolite and its derivatives [35–37].

It has the capacity of acting as an ultraviolet protector, suppressing skin hyperpigmentation and restricting the formation of melanin through the inhibition of tyrosinase formation, an enzyme responsible for pigmentation [38]. And with the prohibition on the use of hydroquinone in the production of cosmetics in several countries, pointed out as a possible carcinogen compound by the Food and Drug Administration (USA), it has led to an expressive increase in the use of kojic acid as a substitute for hydroquinone [39].

In addition to having antioxidant activity and being used in cosmetics intended for skin lightening, kojic acid and its derivatives also have antibiotic properties against certain Gram-negative bacteria, as well as Gram-positive bacteria [40]. Wei et al. [41] pointed to this molecule as a potential drug to be used in corneal transplant patients who suffer from cell senescence and late corneal allograft failure.

Studies also show the antiparasitic effect of this substance. Rodrigues et al. [42], for example, observed that in vitro treatment with a concentration of 50 µg/mL of kojic acid resulted in the activation of mouse peritoneal macrophages, leading to the formation of ROS and a reduction in the number of intracellular *L. amazonensis* amastigotes. In addition, in *in vivo* experiments, it was possible to observe that the topical application of kojic acid promoted the production of type I collagen fibers in the infection sites, as well as a reduction in the size of the skin lesion.

Kojic acid is biologically produced by several fungi during aerobic fermentation, using carbohydrates as a source of energy, and among them, some species of the genus *Aspergillus* stand out, such as *A. oryzae*, *A. parasiticus*, *A. flavus*, *A. tamari*, *A. candidus*, and *A. pseudonomius* [43]. Regarding the production of metabolites, there are several techniques that have been developed and used to optimize fermentation processes, including solid and submerged processes. However, the production of kojic acid reported in the literature is related to submerged fermentation [44].

Even with the valorization of kojic acid, there are no records of industrial production in Brazil. However, several research studies are being carried out for the production of kojic acid from *Aspergillus* species.

## 5. Conclusion

The *Aspergillus* sp. extract isolated from the soil of the Amazon biome showed antimicrobial potential against strains of *S. aureus, S. aureus* MRSA, and *E. coli*, presenting in its composition substances of various chemical classes. The product of this work culminated in the isolation of kojic acid, an unprecedented result for Amazonian fungi, according to the bibliographic survey carried out. This result reinforces the potential of fungi present in the region, demonstrating that they can be viable alternative sources for the production of strategic substances for the industrial sector.

## Figures and Tables

**Table 1:** Antimicrobial activity of *Aspergillus* sp. extract by the cup plate method with inhibition halos in mm.

Fungal extract	Antimicrobial activity (halo size in mm)					
	*E. coli* CBAM	*E. coli* ATCC	*S. aureus* CBAM	*S. aureus* ATCC	*S. aureus* MRSA	C*. albicans* CFAM
*Aspergillus* sp. EtOAc	14	-	10	7	9	-

**Table 2:** Minimum inhibitory concentration of *Aspergillus* sp. extract against test microorganisms in *μ*g/mL.

Fungal Extract/CFAM	Test microorganisms (*μ*g/mL)					
	*E. coli* CBAM	*E. coli* ATCC	*S. aureus* CBAM	*S. aureus* ATCC	*S. aureus* MRSA	C*. albicans* CFAM
*Aspergillus* sp. EtOAc	15.6	-	500	31.2	250	-

**Table 3:** ^1^H,^13^C NMR data of kojic acid (in DMSO-*d*_*6*_: 300 MHz (H) 75 MHz (C)). Literature data obtained in CD_3_OD: 500 MHz (H) 125 MHz (C).

Position	Presented data		Literature data ^*∗*^		
	^13^C (ppm)	^1^H (ppm)	^13^C (ppm)	^1^H (ppm)	HMBC
1	-			-	
2	168.41		170.41		
3	110.23	6.33 *s*, 1H	110.74	6.49 *s*, 1H	C-2, C-3, C-4, C-5, C-7
4	174.33		176.86	-	
5	146.05		147.37		
6	139.68	8.02 *s*, 1H	141.0	7.95 *s*, 1H	C-2, C-4, C-5, C-6
7	59.88	4.29 *s*, 2H	61.18	4.40 *s*, 2H	C-2, C-3, C-7
C7–OH		5.67 *s*, 1H			C-2, C-3, C-7
C5–OH		9.06 *s*, 1H			C-6

^*∗*^The data obtained from DellaGreca et al. [33]. Chemical shift in ppm, coupling constants in Hz. s (singlet).

## Data Availability

The data resulted or analyzed during this study are included in this article. Raw data are available under request.
